# Sagittal Tibial Tunnel Angle Is Associated with Meniscal Extrusion and Clinical Outcomes Following Transtibial Pull-Out Repair of Medial Meniscus Posterior Root Tears

**DOI:** 10.3390/medicina62081596

**Published:** 2026-08-20

**Authors:** Ömer Torun, Merve Kaşıkçı, Ahmet Berkay Girgin, Ahmet Acar, Hüseyin Bilgehan Çevik

**Affiliations:** 1 Department of Orthopedics and Traumatology, Etlik City Hospital, Ankara 06170, Türkiye; acar.ahmet.91@gmail.com (A.A.); bilgehancevik@gmail.com (H.B.Ç.); 2 Department of Biostatistics, Faculty of Medicine, Hacettepe University, Ankara 06100, Türkiye; mervekasikci@outlook.com; 3 Department of Orthopedics and Traumatology, Akyurt State Hospital, Ankara 06750, Türkiye; abgirgin1995@gmail.com

**Keywords:** medial meniscus posterior root tear, meniscal extrusion, sagittal tibial tunnel angle, transtibial pull-out repair, treatment outcome

## Abstract

*Background and Objectives*: This retrospective cohort study aimed to evaluate the association between sagittal tibial tunnel angle and radiological and clinical outcomes following transtibial pull-out repair of medial meniscus posterior root tears. *Materials and Methods*: A total of 81 patients who underwent arthroscopic transtibial pull-out repair between January 2022 and October 2025 and had a minimum follow-up of 6 months were retrospectively evaluated. Demographic, clinical, radiological, and functional data were analyzed. Meniscal extrusion, hip–knee–ankle angle, mechanical axis deviation, sagittal and coronal tunnel angles, International Knee Documentation Committee (IKDC) score, and Numeric Pain Rating Scale (NPRS) score were assessed preoperatively and at 6 months postoperatively. Receiver operating characteristic (ROC) analysis was performed to explore the ability of sagittal tibial tunnel angle to discriminate achievement of the patient acceptable symptom state (PASS) based on the postoperative IKDC score. The ROC-derived 51.5° value was considered an exploratory, cohort-specific threshold. For secondary between-group comparisons, patients were classified into two groups according to sagittal tibial tunnel angle: ≤51.5° and >51.5°. *Results*: The cohort included 56 female patients, and the mean age was 50.0 ± 9.97 years. Receiver operating characteristic analysis identified 51.5° as the exploratory cohort-specific threshold, with an area under the curve of 0.754, sensitivity of 77.8%, and specificity of 68.9%. Patients with a sagittal tunnel angle >51.5° had lower postoperative meniscal extrusion than those with an angle ≤51.5° (3.0 mm versus 4.0 mm, *p* < 0.001), greater reduction in meniscal extrusion (1.10 mm versus 0.3 mm, *p* < 0.001), higher postoperative IKDC (71.62 ± 10.69 versus 53.0 ± 15.44, *p* < 0.001), and lower postoperative NPRS scores (1 versus 4, *p* < 0.001). Achievement of the patient acceptable symptom state was also more frequent in the >51.5° group (66.7% vs. 17.9%, *p* < 0.001). *Conclusions*: A greater sagittal tibial tunnel angle measured on 6-month postoperative MRI was associated with lower concurrent meniscal extrusion, higher functional scores, and lower pain scores following transtibial pull-out repair. However, the retrospective design and short follow-up period limit causal and long-term interpretation. The cohort-derived 51.5° threshold should be considered exploratory and requires external validation before clinical application.

## 1. Introduction

The menisci are essential for load distribution, shock absorption, and maintenance of knee joint stability. The structural integrity of the meniscal roots is essential for converting axial loads into circumferential “hoop” stresses. Disruption of these structures leads to loss of meniscal function, increased tibiofemoral contact pressures, and accelerated osteoarthritic degeneration [1]. In particular, medial meniscus posterior root tears (MMPRTs) have been shown to produce biomechanical consequences comparable to those of total meniscectomy, resulting in rapid degenerative changes in the knee joint [2,3].

Medial meniscus posterior root tears account for approximately 10–21% of all meniscal tears and are most observed in middle-aged and older individuals, often in association with degenerative joint changes [1]. These tears are frequently associated with meniscal extrusion (ME), which disrupts normal load transmission, aggravates cartilage damage, and accelerates osteoarthritis progression [4]. Accordingly, contemporary treatment strategies emphasize the preservation of meniscal tissue and the restoration of the anatomical root attachment [5,6].

The transtibial pull-out technique has emerged as one of the most widely utilized methods for meniscal root repair. This technique aims to restore hoop stress transmission and reduce ME by re-establishing fixation of the meniscal root at its anatomical footprint [7]. Clinical studies have demonstrated that meniscal root repair yields superior functional outcomes and may slow the progression of osteoarthritis compared with meniscectomy [8]. However, it has also been reported that current surgical techniques may not fully restore native meniscal biomechanics, and postoperative healing and ME outcomes remain variable [9]. Lower extremity alignment has been identified as an important factor influencing both the pathogenesis and outcomes of meniscal root tears. Varus alignment and certain tibial morphological characteristics have been associated with MMPRT [3]. In contrast, the impact of surgical variables, especially tibial tunnel positioning and angulation, on the biomechanical effectiveness of repair remains insufficiently characterized.

In the transtibial pull-out technique, variations in tibial tunnel configuration may influence the direction and magnitude of traction applied to the repaired root, potentially affecting postoperative meniscal position and functional recovery [4,7]. Tibial tunnel characteristics can be conceptually separated into two distinct parameters: the position of the intra-articular tunnel aperture, defined by its anteroposterior and mediolateral location relative to the native root attachment, and the sagittal angulation of the tunnel trajectory from the joint surface to the extra-articular tibial exit. The existing literature has focused predominantly on tunnel aperture position. Kamatsuki et al. demonstrated that the accuracy of tibial tunnel aperture placement relative to the anatomical root footprint was associated with meniscal healing and clinical outcomes at 1 year [10], whereas Kawada et al. reported that anatomically accurate tunnel positioning was associated with less progression of medial joint space narrowing and meniscal extrusion at 2 years [11]. These studies primarily characterized where the tunnel aperture was located at the footprint rather than the sagittal trajectory followed by the tunnel. Because tunnel trajectory may vary according to guide orientation, drilling direction, and cortical exit point, tunnels with similar intra-articular aperture positions may have different sagittal angles; conversely, similar sagittal angles may arise from different aperture positions. Tunnel aperture position and sagittal tunnel angle should therefore be considered related but non-interchangeable technical parameters. The clinical relevance of sagittal tibial tunnel angle itself, as distinct from footprint accuracy, remains unclear [12].

Therefore, evaluating the independent effect of sagittal tibial tunnel angulation as distinct from tunnel aperture position on postoperative outcomes is of considerable clinical importance. The aim of this study was to assess the effect of sagittal plane tibial tunnel angle on postoperative ME and clinical outcomes in patients undergoing meniscal root repair using the transtibial pull-out technique.

## 2. Materials and Methods

### 2.1. Study Design and Patient Selection

This retrospective cohort study was conducted between January 2022 and October 2025 at the Department of Orthopaedics and Traumatology, Ankara Etlik City Hospital. Patients were eligible for inclusion if they were aged 15 years or older, had an arthroscopically confirmed medial meniscus posterior root tear, underwent arthroscopic transtibial pull-out repair during the study period, had complete preoperative and postoperative clinical and radiological data, and had a minimum postoperative follow-up of 6 months. All patients were followed for 6 months postoperatively, and clinical and radiological outcomes were assessed at the standardized 6-month follow-up visit. Clinical, radiological, and demographic data for the patients were obtained from the hospital’s electronic medical records system; scales assessing patients’ functional status and pain levels in the preoperative and postoperative periods were completed by the patients in person at the hospital.

**Figure 1:**
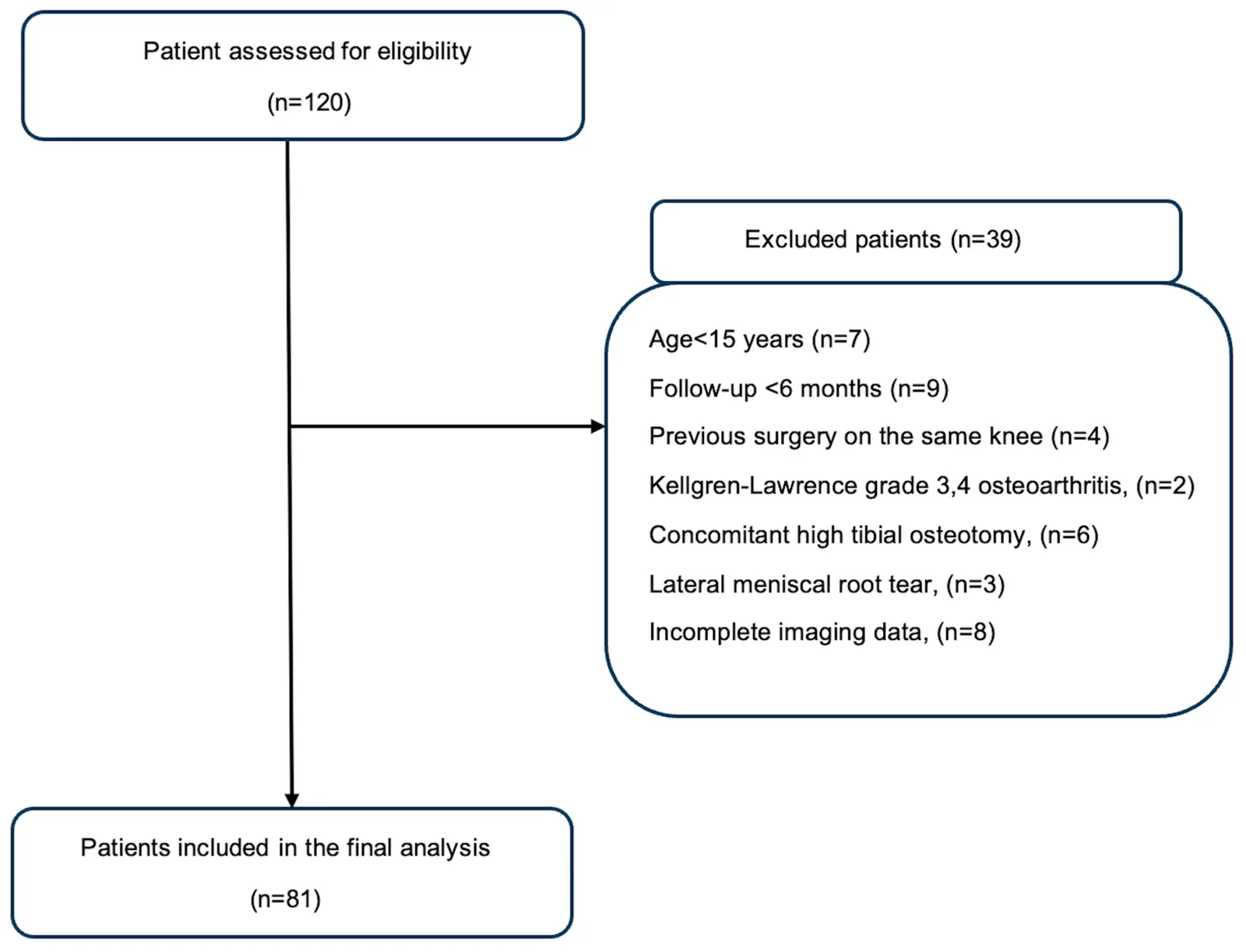
Flow chart of patient selection and study inclusion process.

Patients were excluded if they had a history of previous surgery on the same knee, severe osteoarthritis defined as Kellgren–Lawrence grade 3 or 4 [13], concomitant high tibial osteotomy, a lateral meniscus root tear, infection, tumor, or secondary rheumatological pathology involving the operated knee, or missing required radiological data. The study flow chart is presented in Figure 1.

### 2.2. Clinical and Demographic Variables

Demographic data, including age, sex, and body mass index (BMI), were recorded. Clinical assessment included the side and location of the tear, the presence of concomitant intra-articular knee pathologies, and the type of meniscal root tear. Meniscal root tears were classified according to the LaPrade classification based on intraoperative arthroscopic findings [14]. Knee osteoarthritis severity was graded according to the Kellgren–Lawrence classification using preoperative weight-bearing anteroposterior knee radiographs obtained in the standing position with both knees fully extended [13]. The fixation implant was classified as button fixation, suture anchor fixation, or PushLock anchor fixation.

### 2.3. Radiological Evaluation

In all patients, lower extremity alignment parameters were assessed using long-leg radiographs obtained preoperatively and at the 6th postoperative month. Hip–knee–ankle (HKA) angle, mechanical axis deviation (MAD), medial proximal tibial angle, and mechanical lateral distal femoral angle were measured. To assess measurement reliability, all radiological measurements were independently performed by two observers blinded to clinical outcomes.

MRI acquisition protocol: All knee MRI examinations were performed using a 1.5-T MRI scanner (Gyroscan Intera; Philips Healthcare, Best, The Netherlands) with a dedicated knee coil. The routine knee MRI protocol included axial T1-weighted turbo spin-echo, axial proton density–weighted, axial and coronal fat-suppressed T2-weighted, and sagittal short-tau inversion recovery sequences. The slice thickness was 3 mm. Preoperative and 6-month postoperative MRI examinations were acquired using the same scanner and imaging protocol.

- Hip–knee–ankle (HKA) angle: The HKA angle was calculated in degrees (°) by measuring the angle between the femoral mechanical axis (drawn from the center of the femoral head to the center of the knee) and the tibial mechanical axis (drawn from the center of the knee to the center of the ankle) [15].
- Mechanical axis deviation (MAD): MAD was calculated as the percentage position of the mechanical axis line, drawn from the center of the femoral head to the center of the ankle, relative to the width of the tibial plateau. The medial edge was defined as 0% and the lateral edge as 100%. Values below 50% were considered varus alignment, whereas values above 50% were considered valgus alignment [15].
- Meniscal extrusion (mm) was measured on the mid-coronal MRI slice as the distance between the outer edge of the meniscus and the corresponding edge of the tibial plateau [16].
- The sagittal tibial tunnel angle was measured on sagittal MRI images obtained at the postoperative 6th month as the angle between a line parallel to the joint surface and a line parallel to the tunnel trajectory [17]. Early postoperative MRI was not routinely obtained and was performed only in selected patients with clinical concerns or suspected complications. Because early tunnel-angle measurements were therefore unavailable for the complete cohort and would have represented a clinically selected subgroup, they were not included in the analysis. The present study consequently evaluated the cross-sectional association between sagittal tunnel angle measured at 6 months and concurrent postoperative outcomes.
- The coronal tibial tunnel angle was measured on coronal MRI images as the angle between a line parallel to the joint surface and a line parallel to the tunnel trajectory.
- Sagittal tunnel length (mm) was measured on postoperative 6th month sagittal MRI images as the distance along the longitudinal axis of the tunnel from the intra-articular aperture to the tibial cortical exit [17,18]. Measurements were performed on the slice where the tunnel was most clearly visualized.

### 2.4. Surgical Technique

All procedures were performed by experienced knee surgeons from the same surgical team using a standardized arthroscopic transtibial pull-out repair technique. Following diagnostic arthroscopy, the morphology of the root tear was assessed and classified according to the LaPrade classification. To facilitate healing, the natural attachment site of the meniscal root was debrided using an arthroscopic burr. The meniscus root tear was repaired using the modified Mason–Allen technique [19], using a crescent-shaped suture hook and two reinforced suture fibers. A tibial tunnel was created toward the anatomical root attachment site using an appropriate tibial guide, with a standardized 4 mm diameter for all patients. No predefined sagittal tibial tunnel angle was intentionally targeted intraoperatively. The tunnel trajectory was determined according to the anatomical location of the meniscal root footprint and the feasible positioning of the tibial guide in each patient. The sutures were withdrawn through the tibial tunnel and secured to the tibial cortex using the selected fixation method. Any additional procedures performed as needed were documented. Postoperative rehabilitation was standardized for all patients. Weight-bearing was restricted for the first 4 weeks, and a knee brace was used during this period. Knee range of motion was permitted from 0° to 30° during the first postoperative week and was increased by 30° each week thereafter. Partial weight-bearing was initiated after the fourth postoperative week, and full weight-bearing was allowed at 6 weeks. Return to sports was not assessed in the present study.

### 2.5. Functional and Pain Assessment

Functional outcomes were evaluated using the International Knee Documentation Committee (IKDC) subjective knee score, while pain was assessed using the Numeric Pain Rating Scale (NPRS). These scales were completed by the patients preoperatively and at the postoperative 6th month. Additionally, changes (Δ) for HKA angle, MAD, ME, IKDC score, and NPRS score were calculated as the difference between preoperative and postoperative 6-month values. All clinical, radiological, and functional parameters were also compared between groups according to sagittal tibial tunnel angle.

Clinical outcomes were assessed using the IKDC subjective knee form and the NPRS. The IKDC is a validated patient-reported measure of knee symptoms and function, scored from 0 to 100, with higher scores indicating better outcomes [20]. Pain intensity was assessed using the 11-point NPRS, ranging from 0 (no pain) to 10 (worst imaginable pain) [21].

### 2.6. Ethical Statement

Ethical approval was obtained from the local ethics committee before starting the study approval number: (AESH-BADEK1-2025-766). The study was conducted in accordance with the Declaration of Helsinki. Owing to the retrospective design of the study and the use of anonymized clinical data, the requirement for written informed consent was waived by the Ethics Committee.

### 2.7. Statistical Methods

Statistical analyses were performed using IBM SPSS Statistics v23 (IBM Corp., Armonk, NY, USA) and R Software (version 4.5.2). Because this was a retrospective study and all patients who met the eligibility criteria during the predefined study period were included, no a priori sample size or power calculation was performed. Categorical variables were summarized as frequencies and percentages, while numerical variables were summarized as the mean, standard deviation, minimum, maximum, median, 1st quartile, and 3rd quartile. The distribution of quantitative variables was evaluated using Shapiro–Wilk normality test and graphical methods (histogram and Q-Q plot). For comparisons between two independent groups, the independent-samples t-test was used when the parametric assumptions were satisfied; otherwise, the Mann–Whitney U test was used. Comparisons of numerical variables across more than two independent groups were performed using one-way analysis of variance (ANOVA) or the Kruskal–Wallis test, depending on whether parametric assumptions were met. When a statistically significant difference was detected by the Kruskal–Wallis test, pairwise comparisons were conducted using Dunn’s Bonferroni post hoc test. The association between categorical variables was analyzed using the Pearson chi-square test, Fisher’s exact test, or Fisher–Freeman–Halton test, according to the contingency table structure and expected cell frequencies. To reduce the potential bias arising from multiple comparisons, Benjamini–Hochberg-adjusted *p* values were also presented. Interobserver reliability was assessed using the intraclass correlation coefficient (ICC) based on a two-way random-effects model for absolute agreement and single measurements. Receiver operating characteristic (ROC) curve analysis was performed to explore the discriminative ability of sagittal tibial tunnel angle for achievement of the 6-month IKDC patient acceptable symptom state (PASS), defined as an IKDC score ≥ 67.7 based on a previously published MMPRT-specific threshold [22]. The area under the receiver operating characteristic curve (AUC) was calculated with its 95% confidence interval (CI), and the optimal cutoff value was determined by maximizing the Youden index. Accuracy, sensitivity, specificity, positive predictive value (PPV), negative predictive value (NPV) and F1 score were calculated at the optimal cutoff value. Internal validation of the ROC analysis was performed using 2000 bootstrap resamples while preserving the original numbers of patients in the outcome groups. In each bootstrap resample, the ROC curve was reconstructed and the optimal cutoff value was re-estimated using the Youden index. Optimism was calculated as the difference between the performance obtained in each bootstrap resample and the performance obtained when the bootstrap-derived cutoff was applied to the original dataset. Mean optimism was subtracted from the original-sample estimates to obtain optimism-corrected AUC, accuracy, sensitivity, specificity, PPV, NPV and F1 score. The optimism-corrected performance measures were reported with 95% bootstrap CIs. Cutoff stability was evaluated using the median and interquartile range of the optimal cutoff values obtained across the bootstrap resamples. pROC [23], epiR [24], MLmetrics [25], and boot [26] R packages were used to perform ROC analysis and obtain performance measures with confidence intervals. Multivariable analysis of covariance (ANCOVA) models were fitted to evaluate the association between sagittal plane tunnel angle groups and postoperative outcomes in detail. The models were adjusted for Kellgren–Lawrence grade, the corresponding preoperative outcome value, age, body mass index, operation side, and fixation method. Normality of residuals was evaluated using both statistical test and graphical methods. Multicollinearity among the independent variables was evaluated using variance inflation factor (VIF) values, while heteroscedasticity was assessed using the Breusch–Pagan test. Because the residuals of the postoperative meniscal extrusion and NPRS models did not meet the normality assumption, these outcomes were square-root transformed before analysis. Adjusted estimated marginal means, between-group mean differences, and their 95% confidence intervals were subsequently back-transformed and reported on the original measurement scale. A *p*-value < 0.05 was considered statistically significant.

## 3. Results

Bootstrap internal validation with 2000 resamples yielded an optimism-corrected AUC of 0.754 (95% CI: 0.644–0.855). The median bootstrap-derived cutoff was 51.5 (interquartile range: 51.0–52.2), with 69.0% and 81.0% of cutoff values falling within ±1 and ±2 of 51.5, respectively. These findings supported the central stability of the 51.5 cutoff, although some sampling-related variability was observed (Table 1).

Values are presented as estimates with 95% confidence intervals in parentheses, except for mean optimism. Original-sample performance was calculated in the complete original dataset using the optimal sagittal tibial tunnel angle cutoff value of 51.5°, which was determined by ROC analysis and the Youden index. Internal validation was performed using 2000 bootstrap resamples. In each bootstrap resample, the optimal cutoff was re-estimated, and optimism was calculated as the difference between the performance in the bootstrap resample and the performance obtained when the bootstrap-derived cutoff was applied to the original dataset. Optimism-corrected performance was calculated by subtracting mean optimism from apparent performance. CI, confidence interval; F1 score, harmonic mean of precision and recall; IKDC, International Knee Documentation Committee; NPV, negative predictive value; PASS, patient acceptable symptom state; PPV, positive predictive value; ROC, receiver operating characteristic.

Patients were subsequently classified into ≤51.5° and >51.5° groups for secondary descriptive comparisons.

A total of 81 patients were included in the study, comprising 56 females (69.1%). The mean age was 50.0 ± 9.97 (15–65) years, and the mean body mass index (BMI) was 26.3 ± 3.58 (17.3–33.3). The postoperative follow-up duration was 6 months for all patients. Left meniscal root tears were present in 42 patients (51.8%), and all identified tears involved the posterior meniscal root (*n* = 81, 100%). Concomitant intra-articular knee pathologies were present in 40 patients (49.3%) with osteochondral defect of the medial femoral condyle being the most common (*n* = 26, 32.1%) (Table 2). According to the LaPrade classification, the most common tear type was type 2a (*n* = 46, 56.7%). Based on the Kellgren–Lawrence classification, 54 patients (66.6%) were grade 1, and 27 patients (33.3%) were grade 2.

Anchor fixation was used in 35 patients (43.2%), button implants in 33 patients (40.7%), and push-lock implants in 13 patients (16%). Additional surgical procedures were performed in the same session in 39 patients (48.1%) due to accompanying comorbidities. Postoperative infection requiring debridement occurred in 1 patient (1.2%). Long-leg radiographs revealed a varus deformity in 76 patients (93.8%), and a valgus deformity in 5 patients (6.1%). The mean HKA angle was 4.26° ± 2.65 (−1.8 to 11°) preoperatively and 4.31° ± 2.89 (−2 to 12.1°) at 6 months postoperatively. The mean MAD was 31.3 ± 11.7 (0–58) preoperatively and 31.3 ± 12.9 (−5.2–57) at 6 months postoperatively. The mean medial proximal tibial angle was 86 ± 2.3° (80–89.5°), and the mean mechanical lateral distal femoral angle was 87.8 ± 1.32° (83.2–89.7°). The mean sagittal tunnel angle was 50° ± 9.1 (30–78°) (Table 2).

The mean IKDC score was 42.8 ± 12.2 (22–77) preoperatively and 62.6 ± 16.1 (20–88) at 6 months postoperatively. The median NPRS score was 5 (4–6) preoperatively and 3 (1–5) at 6 months postoperatively.

Interobserver reliability was good to excellent across all radiological measurements, with ICC values ranging from 0.715 to 0.933. Excellent agreement was observed for sagittal tibial tunnel angle (ICC, 0.933; 95% CI, 0.898–0.956), preoperative meniscal extrusion (ICC, 0.920; 95% CI, 0.878–0.948), postoperative meniscal extrusion (ICC, 0.909; 95% CI, 0.862–0.940), medial proximal tibial angle (ICC, 0.911; 95% CI, 0.864–0.942), and mechanical lateral distal femoral angle (ICC, 0.899; 95% CI, 0.847–0.934). Good agreement was observed for preoperative HKA angle, postoperative HKA angle, preoperative MAD, postoperative MAD, coronal tunnel angle, and sagittal tunnel length. All ICC values were statistically significant (all *p* < 0.001).

### Evaluation According to Sagittal Tunnel Angle

No significant differences were observed between the groups in terms of sex, age, BMI, presence of comorbidities, tear localization, type of surgical procedure, complication rates, preoperative HKA angle, varus/valgus alignment, MAD, coronal tunnel angle, medial proximal tibial angle, or mechanical lateral distal femoral angle (*p* > 0.05). Right meniscus involvement was more frequent in the ≤51.5° group, whereas left meniscus involvement was more common in the >51.5° group (*p* = 0.001) (Table 3 and Table 4).

Type 2a tears were more frequent in the >51.5° group, whereas type 2c tears were more frequent in the ≤51.5° group (*p* = 0.004). According to the KL classification, grade 1 osteoarthritis was more common in the >51.5° group, whereas grade 2 was more frequent in the ≤51.5° group (*p* = 0.059) (Table 3).

As a secondary exploratory radiographic analysis, the HKA angle was lower and MAD was higher in the >51.5° group at 6 months postoperatively (*p* = 0.002 and *p* < 0.001, respectively). Given the absence of an alignment-correcting procedure, these differences should not be interpreted as consequences of sagittal tunnel angulation. Sagittal tunnel length was also greater in the >51.5° group (*p* = 0.247). No significant difference was observed between the groups in preoperative ME on MRI (*p* = 0.780); however, postoperative ME was lower in the >51.5° group (*p* < 0.001) (Table 4).

Functional scores were similar between the groups preoperatively, whereas at 6 months postoperatively, they were significantly higher in the >51.5° group (*p* = 0.292, *p* < 0.001). NPRS scores were higher in the >51.5° group preoperatively, whereas they were lower in this group at postoperative 6 months (*p* = 0.029, *p* < 0.001) (Table 4).

When changes (Δ values) were analyzed according to sagittal tunnel angle, between-group differences were observed in ΔHKA and ΔMAD; however, these alignment findings were considered secondary exploratory observations and were not interpreted as consequences of sagittal tunnel angulation. A greater reduction in ME, a greater improvement in IKDC score, and a greater reduction in NPRS score were observed in the >51.5° group (all *p* < 0.001). Detailed comparisons are presented in Table 3 and Table 4.

In the adjusted multivariable ANCOVA models, sagittal tunnel angle group remained significantly associated with all three postoperative outcomes after controlling for Kellgren–Lawrence grade, the corresponding preoperative outcome value, age, BMI, operative side, and fixation method. The adjusted postoperative meniscal extrusion was 4.26 mm (95% CI, 3.95–4.57) in the ≤51.5° group and 3.25 mm (95% CI, 2.99–3.51) in the >51.5° group, with an adjusted mean difference of −1.01 mm (95% CI, −1.43 to −0.59; *p* < 0.001). The adjusted postoperative IKDC scores were 54.76 (95% CI, 50.32–59.20) and 70.01 (95% CI, 65.75–74.27), respectively, with an adjusted mean difference of 15.25 points (95% CI, 8.78–21.72; *p* < 0.001). The adjusted postoperative NPRS scores were 4.25 (95% CI, 3.26–5.24) and 1.88 (95% CI, 1.31–2.45), respectively, with an adjusted mean difference of −2.37 points (95% CI, −3.57 to −1.17; *p* < 0.001) (Table 5).

## 4. Discussion

In this study, the sagittal tibial tunnel angle was significantly associated with both clinical and radiological outcomes following transtibial pull-out repair of posterior root tears of the medial meniscus. In patients with a sagittal tunnel angle > 51.5°, postoperative meniscus extrusion was lower, IKDC scores were higher, and pain scores were lower.

The integrity of the meniscus root attachment is responsible for maintaining circumferential ring tension and ensuring load distribution along the tibiofemoral joint, and is vital for normal knee function. Posterior root tears disrupt this continuity of tension, resulting in a functional outcome equivalent to total meniscectomy [2,27]. Additionally, bone marrow edema caused by the tear has been associated with spontaneous osteonecrosis and accelerated progression of osteoarthritis [28,29,30]. Surgical repair using the transtibial pull-out technique restores anatomical root fixation and yields superior results compared with conservative treatment or partial meniscectomy [31,32]. However, it appears that insufficient attention has been paid to the effect of certain intraoperative parameters, particularly the sagittal tunnel angle, on the quality of root restoration in the transtibial pull-out technique.

Meniscal extrusion is a key radiological indicator of meniscal dysfunction and is closely linked to osteoarthritis progression. Prior studies have reported that ME progresses rapidly following MMPRT [33]. In the present study, overall ME decreased moderately at 6 months postoperatively (3.7 ± 1.2 mm vs. 4.4 ± 0.97 mm), although complete normalization was not achieved, consistent with previous reports [12,34,35]. The reduction in ME was significantly greater in the >51.5° group than in the ≤51.5° group (Δ1.10 mm vs. 0.30 mm, *p* < 0.001). The smaller reduction observed in the low-angle group may be associated with less favorable restoration of hoop stress due to tunnel geometry.

Our findings are consistent with studies reporting favorable average corrections following transtibial pull-out repair [36,37,38,39]. Although many studies report favorable results in terms of average correction, only a limited number of studies have demonstrated statistically significant correction [37]. In the present study, a decrease in ME was observed following the pull-out repair. In contrast to our study, Kawada et al. reported a significant increase in ME following the repair and attributed this to the potential role of the tibial tunnel in promoting the progression of osteoarthritis [12]. However, Higashihara et al. found no significant change in ME during a one-year follow-up in a cohort of 40 patients [40]. A meta-analysis conducted in the United States reported that, compared to partial meniscectomy, the progression of osteoarthritis was less pronounced in transtibial meniscus repair, and ME was lower on postoperative MRI [41]. Variability in ME outcomes may stem from differences in patient selection, follow-up duration, and surgical technique. However, few clinical studies have specifically evaluated the relationship between tibial tunnel orientation and postoperative meniscal extrusion. To the best of our knowledge, this is the first clinical study to evaluate the association between sagittal tibial tunnel angle and postoperative meniscal extrusion following transtibial pull-out repair.

Recent literature increasingly supports the concept that the technical execution of the tibial tunnel, rather than suture configuration or fixation strength alone, may influence structural and clinical outcomes after transtibial pull-out repair. Kwon et al. compared medial and lateral tibial tunnel trajectories and found that tunnel positioning affected the accuracy of anatomical root reduction, although clinical scores did not differ significantly between the groups during follow-up [42]. More directly relevant to tunnel trajectory, Nishino et al. demonstrated in a cadaveric model that an anteriorly positioned tibial tunnel resulted in greater changes in suture length during knee flexion than an anatomically or posteriorly positioned tunnel. They also identified a significant correlation between the anteroposterior tunnel position and the magnitude of suture length change [43]. This finding provides biomechanical plausibility for the present observation that a steeper sagittal tunnel angle, which may alter the line of pull applied to the repaired root, was associated with lower postoperative meniscal extrusion and better functional outcomes. Nevertheless, Nishino et al. evaluated dynamic suture length changes in a cadaveric model rather than postoperative extrusion or clinical outcomes; therefore, the comparison should be regarded as mechanistic rather than directly clinical.

Additional biomechanical evidence indirectly supports the importance of tunnel-related technical factors. In a porcine model, Boksh et al. showed that the addition and positioning of transtibial centralization tunnels influenced meniscal extrusion and tibiofemoral contact mechanics across different knee flexion angles [44]. These findings indicate that tunnel configuration can modify load transmission and meniscal stability rather than functioning merely as a binary anatomical or non-anatomical variable. However, because that study evaluated centralization tunnel configurations rather than sagittal root tunnel orientation, its findings cannot be directly extrapolated to the present angle-based analysis. They nevertheless support the broader principle that tunnel geometry may affect the biomechanical behavior of the repaired meniscus.

Clinical heterogeneity after meniscal root repair remains considerable. A recent systematic review comparing lateral and medial meniscus posterior root repairs reported substantial differences in healing rates, residual extrusion, and clinical outcomes according to root laterality, indicating that anatomical and biomechanical context, rather than tunnel geometry alone, contributes to outcome variability [45]. Similarly, a recent short-term case series demonstrated persistent variability in structural healing and residual extrusion after transtibial pull-out repair and suggested that age and postoperative physiotherapy exposure may influence recovery [46]. Moon et al. further reported that greater meniscal extrusion at 1 year was associated with a lower likelihood of achieving substantial patient-perceived clinical improvement at mid-term follow-up, supporting postoperative meniscal extrusion as a clinically relevant marker rather than a purely radiological finding [47]. Taken together, these observations temper the interpretation of the present results. Although sagittal tibial tunnel angle appears to be a potentially modifiable technical factor associated with postoperative meniscal extrusion and functional recovery, it should be considered one of several interacting determinants, including tunnel aperture position, root tear morphology, osteoarthritis severity, limb alignment, and patient-specific rehabilitation factors.

The associations between sagittal tunnel angle group and postoperative ME, IKDC, and NPRS remained statistically significant after adjustment for Kellgren–Lawrence grade, the corresponding preoperative value, age, BMI, operative side, and fixation method, suggesting that these findings were not explained solely by the measured baseline differences. However, LaPrade tear classification could not be included because of sparse categories, and residual confounding related to tear morphology cannot be excluded. Moreover, the retrospective design precludes causal interpretation.

The lower postoperative ME observed in the >51.5° group may be related to differences in tibial tunnel geometry and the direction of traction applied to the repaired meniscal root. However, this proposed force-vector mechanism was not directly assessed and should therefore be regarded as hypothesis-generating.

The postoperative HKA angle and MAD findings should also be interpreted cautiously. Because no patient underwent an alignment-correcting procedure, these changes are unlikely to represent a direct effect of sagittal tibial tunnel angle and may instead reflect baseline group differences or variability in serial long-leg radiographic measurements. Operative laterality was also unevenly distributed, with right-sided repairs being more frequent in the ≤51.5° group. Although side-dependent effects related to surgeon handedness, standing position, and operative ergonomics have been reported in knee arthroplasty [48,49,50], these factors were not recorded in our cohort. Therefore, operative laterality should be considered a potential unmeasured technical confounder or a chance finding rather than an established explanation.

Furthermore, patients in the >51.5° group had higher postoperative IKDC scores and lower NPRS scores at 6 months. These findings are consistent with previous studies reporting improvements in patient-reported function and pain after transtibial pull-out repair of medial meniscus posterior root tears [38,46,51,52]. However, postoperative recovery is multifactorial and may also be influenced by cartilage degeneration, synovial inflammation, meniscal tissue quality, tear morphology, rehabilitation, and other patient- and surgery-related factors [53,54]. Therefore, sagittal tunnel angle should be interpreted as one potentially relevant variable rather than as an isolated determinant of clinical outcome.

Three fixation methods—suture anchor, button, and PushLock fixation—were used in the present cohort. Although their distribution did not differ significantly between the sagittal tunnel angle groups, fixation technique may theoretically affect construct strength, tension maintenance, healing, and postoperative outcomes. Previous biomechanical studies have reported differing results depending on the fixation construct and experimental conditions [55]. In contrast, a more recent cadaveric study by Cox et al. found that a knotless all-suture anchor construct performed similarly to, or better than, traditional transosseous suture-button fixation in terms of contact area and contact pressure, suggesting that anchor-based fixation may be biomechanically comparable to button fixation under some testing conditions [56]. Because the present study was not designed or adequately powered to compare fixation methods, residual confounding related to fixation technique cannot be completely excluded. Nevertheless, fixation method was included as a covariate in the adjusted multivariable models.

This study has several limitations. First, its retrospective design is a major limitation. Additionally, the 6-month follow-up period permitted assessment of early postoperative radiological and functional outcomes but was insufficient to determine the durability of these findings or to evaluate long-term cartilage preservation and osteoarthritis progression. Although all radiological measurements were performed by two independent and blinded observers, inter-rater variability may still lead to measurement error in continuous parameters such as ME and tunnel angle. The single-center study design may limit the generalizability of these findings to other surgical settings and patient populations.

Adjusted multivariable analyses were performed for postoperative ME, IKDC, and NPRS; however, LaPrade tear classification could not be included because of the very small numbers in some categories and the absence of a clinically appropriate method for combining them. Therefore, residual confounding related to tear morphology cannot be excluded. Although the associations remained significant after adjustment for the available covariates, the retrospective design and concurrent assessment of tunnel angle and outcomes preclude causal interpretation. The absence of a standardized intraoperative protocol for tunnel angle targeting may also have contributed to variation in the sagittal tunnel angle. The significant laterality imbalance between the groups was also unexplained, and surgeon handedness, standing position, portal approach, and side-specific drilling ergonomics were not recorded; therefore, unmeasured technical confounding related to operative side cannot be excluded. Although bootstrap internal validation supported the apparent stability of the cohort-derived 51.5° cutoff and yielded optimism-corrected performance estimates, the threshold has not been externally validated. Therefore, its generalizability remains uncertain, and it should not be used for clinical decision-making or intraoperative targeting until confirmed in an independent patient cohort. Early postoperative MRI was not part of the routine follow-up protocol and was performed only in selected patients who developed clinical concerns or suspected complications. Therefore, early postoperative tunnel-angle measurements were not systematically available for the entire cohort, and restricting the analysis to these patients would have introduced substantial selection by indication. The sagittal tunnel angle was consequently assessed using the routinely obtained 6-month MRI examinations. However, because the tunnel angle and postoperative outcomes were evaluated at the same time point, temporality, directionality, and causality cannot be established. In addition, the 6-month measurement may not precisely represent the original intraoperative tunnel trajectory because potential tunnel remodeling or widening during follow-up cannot be excluded. Accordingly, the findings should be interpreted as concurrent, cross-sectional associations at 6 months Benjamini–Hochberg false discovery rate-adjusted *p* values were calculated for the multiple comparisons in Table 3 and Table 4. Nevertheless, the secondary between-group comparisons should still be interpreted as exploratory. However, to the best of our knowledge, this is one of the first clinical studies to evaluate the association between sagittal tibial tunnel angle and postoperative ME after transtibial pull-out repair.

## 5. Conclusions

Sagittal tibial tunnel angle measured on 6-month postoperative MRI was associated with concurrent meniscal extrusion and short-term patient-reported outcomes following transtibial pull-out repair of medial meniscus posterior root tears. Because of the retrospective design, concurrent assessment of tunnel angle and outcomes, and limited follow-up duration, these findings do not establish causality or long-term clinical significance. The cohort-derived threshold of 51.5° should be regarded as exploratory and hypothesis-generating until externally validated in independent prospective studies with longer follow-up.

## Figures and Tables

**Table 1:** Receiver operating characteristic analysis of sagittal tibial tunnel angle for achievement of the IKDC patient acceptable symptom state.

Performance Measure	Original-Sample Performance	Mean Optimism	Optimism-Corrected Performance
AUC	0.754 (0.644–0.855)	−0.001	0.755 (0.645–0.856)
Accuracy	0.728 (0.618–0.821)	0.026	0.702 (0.617–0.790)
Sensitivity	0.778 (0.608–0.899)	0.027	0.751 (0.639–0.861)
Specificity	0.689 (0.534–0.818)	0.026	0.663 (0.533–0.800)
PPV	0.667 (0.505–0.804)	0.027	0.639 (0.533–0.733)
NPV	0.795 (0.635–0.907)	0.027	0.767 (0.675–0.861)
F1 Score	0.718 (0.615–0.811)	0.027	0.691 (0.603–0.780)

**Table 2:** Demographic, clinical, radiological, and surgical characteristics of the study cohort.

Concomitant Intra-Articular Knee Pathologies, *n* (%)	
Medial femoral condyle osteochondral defect	26 (32.1)
Medial femoral condyle + patella osteochondral defect	5 (6.1)
Trochlea osteochondral defect	5 (6.1)
Medial and lateral femoral condyle osteochondral defect	1 (1.2)
Medial femoral condyle + trochlea osteochondral defect	1 (1.2)
Patella osteochondral defect	1 (1.2)
Lateral femoral condyle osteochondral defect	1 (1.2)
LaPrade classification, *n* (%)	
Type 1	1 (1.2)
Type 2a	46 (56.7)
Type 2b	11 (13.5)
Type 2c	22 (27.1)
Type 3	1 (1.2)
Additional surgical procedures performed, *n* (%)	
Microfracture	23 (28.4)
Microfracture + meniscal centralization	7 (8.6)
Microfracture + MCL release	6 (7.4)
Meniscal centralization	1 (1.2)
MCL release	2 (2.4)
Coronal plane tunnel angle (°)	
Mean ± SD, min–max	70.4 ± 12.6, 13–88.3
Sagittal tunnel length (mm)	
Mean ± SD, min–max	47.8 ± 5.4, 30–61
ME (mm), (Preoperative)	
Mean ± SD, min–max	4.4 ± 0.9, 2.44–7
ME (mm), (postoperative 6 months)	
Mean ± SD, min–max	3.7 ± 1.2, 1.8–7.5

Data are presented as *n* (%) or mean ± standard deviation (minimum–maximum), as appropriate. MCL, medial collateral ligament; ME, meniscal extrusion; SD, standard deviation.

**Table 3:** Comparison of demographic and clinical characteristics according to sagittal plane tunnel angle.

	Sagittal Plane Tunnel Angle			
	≤51.5° (*n* = 39)	>51.5° (*n* = 42)	*p*	q
Sex, *n* (%)				
Male	11 (28.2)	14 (33.3)	0.249 ^a^	
Female	28 (71.8)	28 (66.7)		0.560
Age, years Mean ± SD (min–max)	51.0 ± 9.6 (15–65)	49.1 ± 10.2 (20–63)	0.393 ^d^	0.621
BMI, kg/m^2^ Median (IQR)	26.87 (25.6–28.4)	26.20 (24.1–28.7)	0.590 ^e^	0.621
Laterality, *n* (%)				0.009
Right	26 (66.6)	13 (30.9)	0.001 ^a^	
Left	13 (33.3)	29 (69)		
Concomitant intra-articular pathologies, *n* (%)				
Medial femoral condyle osteochondral defect	14 (63.6)	12 (66.6)	-	
Medial femoral condyle + trochlear osteochondral defect	1 (4.5)	0		
Medial femoral condyle + patellar osteochondral defect	4 (18.1)	1 (5.5)		
Medial + lateral femoral condyle osteochondral defect	0	1 (5.5)		
Trochlear osteochondral defect	3 (14.6)	2 (11.1)		
Patellar osteochondral defect	0	1 (5.5)		
Lateral femoral condyle osteochondral defect	0	1 (5.5)		
LaPrade classification, *n* (%)				0.018
Type 1	1 (2.5)	0	0.004 ^c^	
Type 2a	21 (53.8)	25 (59.5)		
Type 2b	1 (2.5)	10 (23.8)		
Type 2c	15 (38.4)	7 (16.6)		
Type 3	1 (2.5)	0		
Kellgren–Lawrence classification, *n* (%)				
Grade 1	22 (56.4)	32 (76.2)	0.059 ^a^	0.177
Grade 2	17 (43.6)	10 (23.8)		
Meniscal tear location, *n* (%)				0.621
Anterior root	0	0	0.481 ^b^	
Posterior root	39 (100)	42 (100)		
Type of surgical implant, *n* (%)				
PushLock anchor	6 (15.3)	7 (16.6)	0.621 ^a^	0.621
Suture anchor	15 (38.4)	20 (47.6)		
Button implant	18 (46.1)	15 (35.7)		
Complications, *n* (%)	0	2(4.5)	0.498 ^b^	0.621

Data are presented as *n* (%), mean ± standard deviation (minimum–maximum), or median (IQR), as appropriate. ^a^: Pearson Chi-square test, ^b^: Fisher’s exact test, ^c^: Fisher–Freeman–Halton test, ^d^: Independent samples *t*-test, ^e^: Mann–Whitney U test, BMI, body mass index; IQR, interquartile range; SD, standard deviation; q, Benjamini–Hochberg false discovery rate–adjusted *p* value.

**Table 4:** Comparison of radiological and functional outcomes according to sagittal plane tunnel angle.

	Sagittal Plane Tunnel Angle			
	≤51.5° (*n* = 39)	>51.5° (*n* = 42)	*p*	q
Type of knee alignment deformity, *n* (%)				
Varus	38 (97.4)	38 (90.5)	0.361 ^b^	0.399
Valgus	1 (2.5)	4 (9.5)		
HKA angle (°), preoperative Mean ± SD (min–max)	4.6 ± 2.6 (−1.8–10.3)	3.9 ± 2.7 (−1.80–11)	0.220 ^d^	0.289
HKA angle (°), postoperative 6 months Mean ± SD (min–max)	5.3 ± 2.8 (−1–12.1)	3.9 ± 2.7 (−2–10)	0.002 ^d^	0.004
MAD (%), preoperative Mean ± SD (min–max)	28.7 ± 11.6	33.7 ± 11.5	0.055 ^d^	0.089
MAD (%), postoperative 6 months Mean ± SD (min–max)	25.78 ± 12.9 (−5.26–50)	36.43 ± 10.8 (12.60–57)	<0.001 ^d^	<0.001
Medial proximal tibial angle (°) Median (IQR)	85.5 (84–87)	87 (85–88)	0.079 ^e^	0.118
Mechanical lateral distal femoral angle (°), Median (IQR)	88 (87–89)	88.1 (87.2–89)	0.747 ^e^	0.780
Coronal plane tunnel angle (°) Median (IQR)	68 (62–80)	74 (67.8–80)	0.120 ^e^	0.168
Sagittal tunnel length (mm) Mean ± SD (min–max)	47.1 ± 5.83 (30–61)	48.5 ± 4.95 (35–58)	0.247 ^d^	0.305
Preoperative MRI ME (mm) Median (IQR)	4.5 (3.7–5)	4 (3.8–5)	0.780 ^e^	0.780
Postoperative MRI ME (mm) Median (IQR)	4 (3.6–5)	3 (2.7–3.4)	<0.001 ^e^	<0.001
IKDC score, preoperative Mean ± SD (min–max)	41.2 ± 12.8 (22–77)	44.2 ± 11.6 (24–72)	0.292 ^d^	0.341
IKDC score, postoperative 6 months Mean ± SD (min–max)	53 ± 15.4 (20–82)	71.6 ± 10.7 (48–88)	<0.001 ^d^	<0.001
IKDC score, preoperative, *n* (%) <67.7 (PASS not achieved) ≥67.7 (PASS achieved)	38 (97.4) 1 (2.5)	41 (97.6) 1 (2.3)	-	
IKDC score, postoperative 6 months, *n* (%) <67.7 (PASS not achieved) ≥67.7 (PASS achieved)	31 (79.4) 8 (20.6)	14 (33.3) 28 (66.6)	<0.001 ^a^	<0.001
NPRS score, preoperative Median (IQR)	5 (3–5)	5 (4–6)	0.029 ^e^	0.051
NPRS score, postoperative 6 months Median (IQR)	4 (3–6)	1 (0–3)	<0.001 ^e^	<0.001
Δ HKA angle (°) Median (IQR)	−0.8 (−1.6–0.6)	0.6 (−0.1–1.3)	<0.001 ^e^	<0.001
Δ MAD Median (IQR)	4 (−2–6)	−2.75 (−6–0.7)	<0.001 ^e^	<0.001
Δ ME (mm) Median (IQR)	0.3 (−0.6–0.8)	1.10 (0.7–1.6)	<0.001 ^e^	<0.001
Δ IKDC score (Mean ± sd, min–max)	−11.7 ± 17.3 (−48–22)	−27.4 ± 15.7 (−55–10)	<0.001 ^d^	<0.001
Δ NPRS score Median (IQR)	0 (−1–2)	4 (2–5)	<0.001 ^e^	<0.001

Data are presented as *n* (%), mean ± standard deviation (minimum–maximum), or median (IQR), as appropriate, according to the distribution of the variables within the comparison groups. ^a^: Pearson chi-square test; ^b^: Fisher’s exact test; ^d^: independent samples *t*-test; ^e^: Mann–Whitney U test. HKA, hip–knee–ankle; IKDC, International Knee Documentation Committee; IQR, interquartile range; MAD, mechanical axis deviation; ME, Meniscal extrusion; MRI, magnetic resonance imaging; NPRS, Numeric Pain Rating Scale; PASS, patient-acceptable symptomatic state; q, Benjamini–Hochberg false discovery rate–adjusted *p* value; SD, standard deviation; Δ: preoperative value–postoperative 6-month value.

**Table 5:** Adjusted postoperative outcomes according to sagittal tibial tunnel angle group.

	Adjusted Means			
	Sagittal Plane Tunnel Angle ≤ 51.5°	Sagittal Plane Tunnel Angle > 51.5°	Adjusted Mean Differences	*p*-Value
Postop_ME	4.26 (3.95–4.57)	3.25 (2.99–3.51)	−1.01 (−1.43–−0.59)	<0.001
Postop_IKDC	54.76 (50.32–59.20)	70.01 (65.75–74.27)	15.25 (8.78–21.72)	<0.001
Postop_NPRS	4.25 (3.26–5.24)	1.88 (1.31–2.45)	−2.37 (−3.57–−1.17)	<0.001

Values are presented as adjusted estimated marginal means with 95% confidence intervals. Adjusted mean differences were calculated as the >51.5° group minus the ≤51.5° group. Separate multivariable analysis of covariance models were fitted for each postoperative outcome and adjusted for Kellgren–Lawrence grade, the corresponding preoperative outcome value, age, body mass index, operative side, and fixation method. Postoperative meniscal extrusion and NPRS were square-root transformed because of non-normal residuals; adjusted estimates and 95% confidence intervals were subsequently back-transformed and are presented on the original measurement scales. IKDC, International Knee Documentation Committee; ME, meniscal extrusion; NPRS, Numeric Pain Rating Scale.

## Data Availability

The data supporting the findings of this study are not publicly available due to privacy and ethical restrictions. The data may be made available from the corresponding author upon reasonable request and with permission from the relevant institutional authorities.

## Abbreviations

AUC	Area under the receiver operating characteristic curve
BMI	Body mass index
CI	Confidence interval
HKA	Hip–knee–ankle
ICC	Intraclass correlation coefficient
IKDC	International Knee Documentation Committee
IQR	Interquartile range
KL	Kellgren–Lawrence
MAD	Mechanical axis deviation
MCL	Medial collateral ligament
ME	Meniscal extrusion
MMPRT	Medial meniscus posterior root tear
MRI	Magnetic resonance imaging
NPV	Negative predictive value
NPRS	Numeric Pain Rating Scale
PASS	Patient-acceptable symptomatic state
PPV	Positive predictive value
ROC	Receiver operating characteristic
SD	Standard deviation

## References

[1] Bhatia S., LaPrade C.M., Ellman M.B., LaPrade R.F. (2014). Meniscal root tears: Significance, diagnosis, and treatment. Am. J. Sports Med..

[2] Allaire R., Muriuki M., Gilbertson L., Harner C.D. (2008). Biomechanical consequences of a tear of the posterior root of the medial meniscus: Similar to total meniscectomy. JBJS.

[3] Oeding J.F., Dean M.C., Hevesi M., Chahla J., Krych A.J. (2025). Steeper slope of the medial tibial plateau, greater varus alignment, and narrower intercondylar distance and notch width increase risk for medial meniscus posterior root tears: A systematic review. Arthroscopy.

[4] Graham B.C., Weiss-Laxer N.S., Haider M.N., Marzo J.M. (2024). Item-specific knee injury and osteoarthritis outcome score characterization of patients with medial meniscus root tear. Orthop. J. Sports Med..

[5] Cabarcas B.C., Ina J., Ward G.H., Hevesi M., Krych A.J. (2025). Meniscus Root Injuries: Modern Techniques and Clinical Outcomes. Oper. Tech. Orthop..

[6] Homan M.D., Braaten J.A., Banovetz M.T., Kennedy N.I., LaPrade R.F. (2023). Meniscal root tears: Repair and salvage techniques. J. Cartil. Jt. Preserv..

[7] Garcia J.R., Ayala S.G., Allende F., Mameri E., Haynes M., Familiari F., Geeslin A.G., Murray I., Moatshe G., Verma N.N. (2024). Diagnosis and treatment strategies of meniscus root tears: A scoping review. Orthop. J. Sports Med..

[8] Tamura M., Furumatsu T., Yokoyama Y., Higashihara N., Kawada K., Ozaki T. (2024). Superior outcomes of pullout repairs for medial meniscus posterior root tears in partial tear compared to complete radial tear. Knee Surg. Relat. Res..

[9] Liao X., Li H., Nie S., Lan M. (2023). Risk factors of incomplete healing following medial meniscus posterior root tear repair with gracilis tendon. Sci. Rep..

[10] Kamatsuki Y., Furumatsu T., Hiranaka T., Okazaki Y., Kodama Y., Kintaka K., Ozaki T. (2021). Accurate placement of a tibial tunnel significantly improves meniscal healing and clinical outcomes at 1 year after medial meniscus posterior root repair. Knee Surg. Sports Traumatol. Arthrosc..

[11] Kawada K., Okazaki Y., Tamura M., Yokoyama Y., Ozaki T., Furumatsu T. (2024). Accurate tibial tunnel position in transtibial pullout repair for medial meniscus posterior root tears delays the progression of medial joint space narrowing. Knee Surg. Sports Traumatol. Arthrosc..

[12] Kawada K., Furumatsu T., Tamura M., Xue H., Higashihara N., Kintaka K., Yokoyama Y., Ozaki T. (2023). Medial joint space narrowing progresses after pullout repair of medial meniscus posterior root tear. Int. Orthop..

[13] Kellgren J.H., Lawrence J. (1957). Radiological assessment of osteo-arthrosis. Ann. Rheum. Dis..

[14] Feagin J.A., Engebretsen L., Cram T.R., James E.W., LaPrade C.M., LaPrade R.F. (2015). Meniscal root tears: A classification system based on tear morphology. Am. J. Sports Med..

[15] Moreland J.R., Bassett L., Hanker G. (1987). Radiographic analysis of the axial alignment of the lower extremity. J. Bone Jt. Surg..

[16] Costa C.R., Morrison W.B., Carrino J.A. (2004). Medial meniscus extrusion on knee MRI: Is extent associated with severity of degeneration or type of tear?. Am. J. Roentgenol..

[17] LaPrade R.F., Matheny L.M., Moulton S.G., James E.W., Dean C.S. (2017). Posterior meniscal root repairs: Outcomes of an anatomic transtibial pull-out technique. Am. J. Sports Med..

[18] Röpke E.F., Kopf S., Drange S., Becker R., Lohmann C.H., Stärke C. (2015). Biomechanical evaluation of meniscal root repair: A porcine study. Knee Surg. Sports Traumatol. Arthrosc..

[19] Lee D.W., Jang S.H., Ha J.K., Kim J.G., Ahn J.H. (2013). Meniscus root refixation technique using a modified Mason–Allen stitch. Knee Surg. Sports Traumatol. Arthrosc..

[20] Çelik D., Coşkunsu D., Kılıçoğlu Ö., Ergönül Ö., Irrgang J.J. (2014). Translation and cross-cultural adaptation of the international knee documentation committee subjective knee form into Turkish. J. Orthop. Sports Phys. Ther..

[21] Jensen M.P., McFarland C.A. (1993). Increasing the reliability and validity of pain intensity measurement in chronic pain patients. Pain.

[22] Garcia J.R., Allende F., Atkins M.A., McCormick J.R., Yanke A.B., Cole B.J., Verma N.N., Chahla J. (2025). Patient-reported outcomes measurement information system captures clinically meaningful improvement after transtibial pull-out repair of medial meniscal posterior root tears: 2-year outcomes. Arthroscopy.

[23] Robin X., Turck N., Hainard A., Tiberti N., Lisacek F., Sanchez J.-C., Müller M. (2011). pROC: An open-source package for R and S+ to analyze and compare ROC curves. BMC Bioinform..

[24] Stevenson M., Sergeant E., Nunes T., Heuer C., Marshall J., Sanchez J., Thornton R., Reiczigel J., Robison-Cox J., Sebastiani P. (2018). epiR: Tools for the Analysis of Epidemiological Data.

[25] Yan Y. (2015). MLmetrics: Machine learning evaluation metrics. CRAN: Contributed Packages. https://CRAN.R-project.org/package=MLmetrics.

[26] Loh W.W. (2026). Leveraging lavaan for iterated conditional expectations: A streamlined parametric g-formula. Psychol. Methods.

[27] Marzo J.M., Gurske-DePerio J. (2009). Effects of medial meniscus posterior horn avulsion and repair on tibiofemoral contact area and peak contact pressure with clinical implications. Am. J. Sports Med..

[28] Kattapuram T.M., Kattapuram S.V. (2008). Spontaneous osteonecrosis of the knee. Eur. J. Radiol..

[29] Robertson D., Armfield D., Towers J., Irrgang J., Maloney W., Harner C. (2009). Meniscal root injury and spontaneous osteonecrosis of the knee: An observation. J. Bone Jt. Surg. Br. Vol..

[30] Sung J.H., Ha J.K., Lee D.W., Seo W.Y., Kim J.G. (2013). Meniscal extrusion and spontaneous osteonecrosis with root tear of medial meniscus: Comparison with horizontal tear. Arthroscopy.

[31] Padalecki J.R., Jansson K.S., Smith S.D., Dornan G.J., Pierce C.M., Wijdicks C.A., LaPrade R.F. (2014). Biomechanical consequences of a complete radial tear adjacent to the medial meniscus posterior root attachment site: In situ pull-out repair restores derangement of joint mechanics. Am. J. Sports Med..

[32] Woodmass J.M., LaPrade R.F., Sgaglione N.A., Nakamura N., Krych A.J. (2017). Meniscal repair: Reconsidering indications, techniques, and biologic augmentation. JBJS.

[33] Furumatsu T., Kodama Y., Kamatsuki Y., Hino T., Okazaki Y., Ozaki T. (2017). Meniscal extrusion progresses shortly after the medial meniscus posterior root tear. Knee Surg. Relat. Res..

[34] Chung K.S., Ha J.K., Ra H.J., Kim J.G. (2016). A meta-analysis of clinical and radiographic outcomes of posterior horn medial meniscus root repairs. Knee Surg. Sports Traumatol. Arthrosc..

[35] Moon H.-K., Koh Y.-G., Kim Y.-C., Park Y.-S., Jo S.-B., Kwon S.-K. (2012). Prognostic factors of arthroscopic pull-out repair for a posterior root tear of the medial meniscus. Am. J. Sports Med..

[36] Jung Y.-H., Choi N.-H., Oh J.-S., Victoroff B.N. (2012). All-inside repair for a root tear of the medial meniscus using a suture anchor. Am. J. Sports Med..

[37] Kim S.B., Ha J.K., Lee S.W., Kim D.W., Shim J.C., Kim J.G., Lee M.Y. (2011). Medial meniscus root tear refixation: Comparison of clinical, radiologic, and arthroscopic findings with medial meniscectomy. Arthroscopy.

[38] Lee J.H., Lim Y.J., Kim K.B., Kim K.H., Song J.H. (2009). Arthroscopic pullout suture repair of posterior root tear of the medial meniscus: Radiographic and clinical results with a 2-year follow-up. Arthroscopy.

[39] Sundararajan S.R., Ramakanth R., Sethuraman A.S., Kannan M., Rajasekaran S. (2022). Correlation of factors affecting correction of meniscal extrusion and outcome after medial meniscus root repair. Arch. Orthop. Trauma Surg..

[40] Higashihara N., Furumatsu T., Okazaki Y., Yokoyama Y., Tamura M., Kawada K., Hasegawa T., Kohara T., Ozaki T. (2024). Transtibial pullout repair improved short-term clinical outcomes in patients with oblique medial meniscus posterior root tear comparable to radial root tear. Eur. J. Orthop. Surg. Traumatol..

[41] Faucett S.C., Geisler B.P., Chahla J., Krych A.J., Kurzweil P.R., Garner A.M., Liu S., LaPrade R.F., Pietzsch J.B. (2019). Meniscus root repair vs meniscectomy or nonoperative management to prevent knee osteoarthritis after medial meniscus root tears: Clinical and economic effectiveness. Am. J. Sports Med..

[42] Kwon S.-W., Kim J.B., Kim C.H., Hong S.J., Hong Y.C., Jang B.-W. (2020). Comparison of medial and lateral tibial tunnel in pullout repair of posterior root tear of medial meniscus: Radiologic, clinical, and arthroscopic outcomes. J. Orthop. Surg..

[43] Nishino K., Hashimoto Y., Kinoshita T., Iida K., Tsumoto S., Nakamura H. (2024). Anterior bone tunnel position increases meniscus migration in medial meniscus posterior root repair: A cadaveric study of suture length changes. J. Exp. Orthop..

[44] Boksh K., Espino D.M., Ghosh A., Aujla R., Boutefnouchet T., Shepherd D.E. (2025). Transtibial centralization better restores meniscal extrusion and contact mechanics compared with knotless anchor centralization for medial meniscal posterior root tears: An in vitro biomechanical study using a porcine model. Arthrosc. J. Arthrosc. Relat. Surg..

[45] Dzidzishvili L., Pedemonte-Parramón G., Garcia-Oltra E., López V., Hernández-Hermoso J.A. (2025). Lateral meniscus posterior root repairs show superior healing, reduced meniscal extrusion and improved clinical outcomes compared to medial meniscus posterior root repairs: A systematic review. Knee Surg. Sports Traumatol. Arthrosc..

[46] Nistor D.V., Piu S., Mihu D.R., von Mengershausen R. (2025). Short-Term Outcomes After Transtibial Repair of Medial Meniscus Posterior Root Tears: A Case Series. J. Clin. Med..

[47] Moon H.S., Jung M., Choi C.H., Chung K., Jung S.H., Byun J., Ham H., Kim S.H. (2025). Increased meniscal extrusion at 1 year after surgery is associated with a lower likelihood of substantial mid-term patient-perceived improvement after medial meniscal root tear repair. Arthroscopy.

[48] Mehta S., Lotke P.A. (2007). Impact of surgeon handedness and laterality on outcomes of total knee arthroplasties: Should right-handed surgeons do only right TKAs?. Am. J. Orthop..

[49] Hernigou P., Scarlat M.M. (2026). Left-handed orthopaedic surgeons, left-sided patients, and left-threaded screw extractors in a right-handed world: Understanding the largest minority, the dominant side, the laterality, and situs inversus in orthopaedics. Int. Orthop..

[50] Cao Z., Liu Y., Yang M., Zhang Z., Kong X., Chai W. (2022). Effects of surgeon handedness on the outcomes of unicompartmental knee arthroplasty: A single center’s experience. Orthop. Surg..

[51] Cortina G., Perelli S., Pizza N., Condello V., Madonna V., Monllau J.C. (2026). Medial meniscus posterior root tear repair: An overview of surgical techniques and augmentation strategies. J. Exp. Orthop..

[52] Tageldein M.M., Alhamdah Y., Bouchard M.D., Vivekanantha P., Murphy B., Hoshino Y., Lesniak B., de Sa D. (2026). Postoperative rehabilitation after transtibial pullout repair of medial meniscus posterior root tears: A systematic review and meta-analysis. Knee Surg. Sports Traumatol. Arthrosc..

[53] Olivotto E., Belluzzi E., Pozzuoli A., Cigolotti A., Scioni M., Goldring S.R., Goldring M.B., Ruggieri P., Ramonda R., Grigolo B. (2022). Do synovial inflammation and meniscal degeneration impact clinical outcomes of patients undergoing arthroscopic partial meniscectomy? A histological study. Int. J. Mol. Sci..

[54] Olivotto E., Trisolino G., Belluzzi E., Lazzaro A., Strazzari A., Pozzuoli A., Cigolotti A., Ruggieri P., Evangelista A., Ometto F. (2022). Macroscopic synovial inflammation correlates with symptoms and cartilage lesions in patients undergoing arthroscopic partial meniscectomy: A clinical study. J. Clin. Med..

[55] Wu S.-H., Yeh T.-T., Hsu W.-C., Wu A.T., Li G., Chen C.-H., Lee C.-H., Wu J.-L. (2020). Biomechanical comparison of four tibial fixation techniques for meniscal root sutures in posterior medial meniscus root repair: A porcine study. J. Orthop. Transl..

[56] Cox K.G., Weldy J.M., Beason D.P., MacDonell R.T., Fleisig G.S., Vega T.F., Cain E.L. (2026). Biomechanical Comparison of Knotless Suture Anchor Fixation Versus Transosseous Suture Button Fixation for Posterior Medial Meniscal Root Tears. Orthop. J. Sports Med..

